# The Development of a Portable SPR Bioanalyzer for Sensitive Detection of *Escherichia coli* O157:H7

**DOI:** 10.3390/s16111856

**Published:** 2016-11-04

**Authors:** Shun Wang, Jiufeng Xie, Min Jiang, Keke Chang, Ruipeng Chen, Liuzheng Ma, Juanhua Zhu, Qingqian Guo, Haifeng Sun, Jiandong Hu

**Affiliations:** 1 Department of Electrical Engineering, Henan Agricultural University, Zhengzhou 45002, China; wangshun6518@163.com (S.W.); changkeke927@163.com (K.C.); chen_ruipeng@yeah.net (R.C.); mlz0124@126.com (L.M.); zhujh88@sina.com (J.Z.); guo_qingqian@163.com (Q.G.); haifengdreams@sina.cn (H.S.); 2 State Key Laboratory of Wheat and Maize Crop Science, Zhengzhou 45002, China; 3 College of life sciences, Henan Agricultural University, Zhengzhou 45002, China; xiejiufeng0809@126.com (J.X.); jm_hu@163.com (M.J.)

**Keywords:** *Escherichia coli* O157:H7, portable surface plasmon resonance (SPR) bioanalyzer, biomolecular recognition membrane, ELISA kit, response unit

## Abstract

The purpose of this study was to develop a portable surface plasmon resonance (SPR) bioanalyzer for the sensitive detection of *Escherichia coli* O157:H7 in comparison with an enzyme-linked immunosorbent assay (ELISA). The experimental setup mainly consisted of an integrated biosensor and a homemade microfluidic cell with a three-way solenoid valve. In order to detect *Escherichia coli* O157:H7 using the SPR immunoassay, 3-mercaptopropionic acid (3-MPA) was chemisorbed onto a gold surface via covalent bond for the immobilization of biological species. 1-ethyl-3-(3-dimethylaminopropyl) carbodiimide hydrochloride (EDC) and N-hydroxysuccinimide (NHS) were used as crosslinker reagents to enable the reaction between 3-MPA and *Escherichia coli* O157:H7 antibodies by covalent –CO–NH– amide bonding. The experimental results were obtained from the *Escherichia coli* O157:H7 positive samples prepared by 10-, 20-, 40-, 80-, and 160-fold dilution respectively, which show that a good linear relationship with the correlation coefficient R of 0.982 existed between the response units from the portable SPR bioanalyzer and the concentration of *Escherichia coli* O157:H7 positive samples. Moreover, the theoretical detection limit of 1.87 × 10^3^ cfu/mL was calculated from the positive control samples. Compared with the *Escherichia coli* O157:H7 ELISA kit, the sensitivity of this portable SPR bioanalyzer is four orders of magnitude higher than the ELISA kit. The results demonstrate that the portable SPR bioanalyzer could provide an alternative method for the quantitative and sensitive determination of *Escherichia coli* O157:H7 in field.

## 1. Introduction

For the prevention of infection, illness, and economic loss, rapid detection of pathogenic microorganisms in food and water has been a challenge for decades. Most food poisoning cases are caused by microbial pollution, where *Escherichia coli (E. coli)* is one of the most common pathogenic bacteria and brings great harm to human body health. Furthermore, as is widely known to the public, *E. coli* O157:H7 can cause human enteritis and hemorrhagic diarrhea. Annual economic losses due to human norovirus, *E. coli* O157:H7, have been estimated to be $0.27 billion [1]. Hence, some novel, rapid, and more practical techniques for the identification of bacterial contamination in food are urgently required.

Recently, a variety of techniques have been developed for the identification of *E. coli* O157:H7, including serological separation, bacteria morphology, and flow cytometry [2,3]. Although these techniques have been successfully utilized to detect the *E. coli* O157:H7 in food samples, some of these methods require sophisticated and expensive instruments, or radioactive and expensive chemicals, or involve a tedious procedure. Moreover, it is difficult for some methods to be used for rapid detection and quantification of the *E. coli* O157:H7 at low concentrations due to the complicated preparation. For instance, the method of microbiological culture needs to take one or more enrichment cycles and work up to 36 h to obtain the measurement results. Therefore, it is of considerable interest to explore a low-cost approach for the identification of *E. coli* O157:H7. Serological classification is used for the detection of *E. coli* O157:H7 mainly by using a monoclonal antibody to establish an enzyme-linked immunosorbent assay (ELISA) and the latex coagulation experiment [4]. Immunologic-based biosensor technologies, such as amperometricimmuno sensors [4,5,6,7], electrochemical detection [8,9], piezoelectric biosensors [10,11], electrical impedance biosensors [12], and immunomagnetic biosensors [13] have been widely applied for the detection of bacteria. With the rapid development in molecular biology, the polymerase chain reaction (PCR) has been widely used to evaluate *E. coli* O157:H7 without environmental influences [14,15,16]. A successful combination of physics and biology, the low cost surface plasmon resonance (SPR) biosensor has been developed in recent years. With the advantages of label-free, non-intrusive, real-time biomolecule detection, SPR biosensors have been widely applied in life science, drug development, environmental monitoring, and food safety [17,18,19]. An increasing number of research groups have been dedicated to studying the detection of the pathogens, such as *Staphylococcus aureus*, bacteriophages, and *E. coli* O157:H7 using quartz crystal microbalance (QCM) and the electrochemical method [20,21,22,23,24]. Compared with the QCM and the electrochemical method, SPR has some prominent advantages: firstly, it can achieve real time monitoring of biomolecular interactions; secondly, it can determine both kinetics parameters. However, an enzyme-linked immunosorbentassay (ELISA) kit is one frequently used method to detect *E. coli* O157:H7 in situ, which combines the specificity of antibodies with the sensitivity of simple enzyme assays by using antibodies or antigens coupled to an easily-assayed enzyme. However, in most cases, such as meat and dairy products, the concentration of *E. coli* O157:H7 is too low, which frequently leads to a false positive result [25]. To handle this problem, label-free, non-intrusive, real-time biomolecule detection was introduced, which is based on a low-cost SPR biosensor combined with microfluidic cell techniques.

The aim of the present investigation is to develop a portable SPR bioanalyzer to monitor the specific binding reaction between *E. coli* O157:H7 antigen and antibody. The *E. coli* O157:H7 antibody was covalently attached on the 3-MPA/Au SPR chip, and the detection of *E. coli* O157:H7 was conducted. A sensitive SPR bioanalyzer was provided to greatly realize the label-free and real-time *E. coli* O157:H7 antigen and antibody analysis. To verify the performance of the portable SPR bioanalyzer, the ELISA kit was also used for the detection of *E. coli* O157:H7. By comparing the experiment results obtained from these two approaches, the fabricated portable SPR bioanalyzer shows great potential for rapid detection of *E. coli* O157:H7.

## 2. Materials and Methods

### 2.1. Materials

An *Escherichia coli* O157:H7 ELISA kit and the positive control sample containing 4.0 × 10^5^ cfu/mL of *E. coli* O157:H7 were purchased from Biolab Co., Ltd. (Beijing, China). The positive control sample containing 4 × 10^5^ cfu/mL of *E. coli* O157:H7 was firstly diluted to 4.0 × 10^4^ cfu/mL (10-fold step). Then, the positive control sample containing 4 × 10^4^ cfu/mL of *E. coli* O157:H7 was serially diluted to 0.25 × 10^4^–2.0 × 10^4^ cfu/mL (2-fold steps) for the measurement.

3-Mercaptopropionic acid (MPA), ethanol amine (Eth), 1-ethyl-(3-3-dimethylaminopro-pyl) carbodiimide hydrochloride (EDC), N-hydroxysuccinimide (NHS), phosphate buffer (PBS) (pH 7.4), NaOH, and sodium dodecyl sulfonate (SDS) were purchased from Shanghai General Chemical Reagent Factory (Shanghai, China). The integrated biosensor modular (Spreeta biosensor) manufactured with a 50 nm gold slide bonded to the sensor modules was purchased from Nomadics, Inc. (Stillwater, OK, USA).

### 2.2. Methods

**Figure 1:**
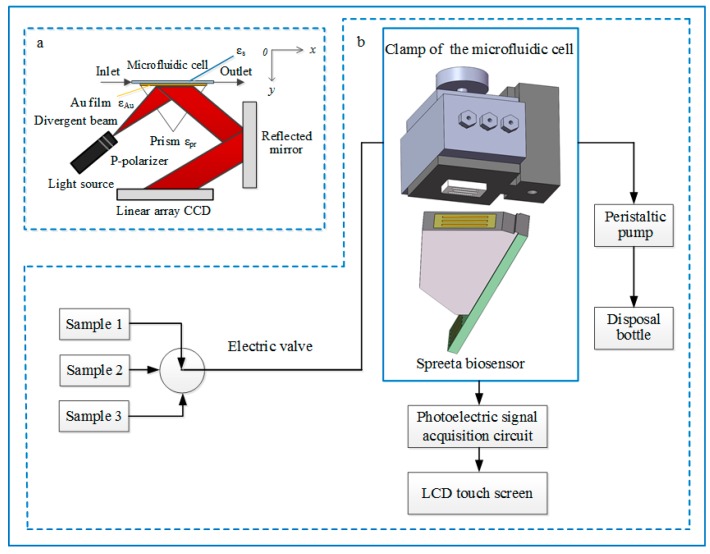
The schematic diagram of the portable surface plasmon resonance (SPR) bioanalyzer. (**a**): Principle of surface plasmon resonance biosensing platform; (**b**): Schematic representation of construction of the portable SPR bioanalyzer.

The surface plasmon wave (SPW) at the metal–dielectric interface, decaying evanescently into both media, is derived from the electron density oscillations caused by a TM (transverse magnetic) or p-polarized light. This TM field intensity makes the surface plasmon in the vicinity of the surface of the metal (<100 nm) very sensitive to the sample mass. The perturbations of the SPW are highly sensitive to the small changes in refractive index (RI). For the SPR bioanalyzer constructed by SPR biosensors using the coupling method of the attenuated total reflection (ATR), the propagation constants of the incident light wave and the SPW along the x-axis are the same (see Figure 1). In this case, the resonance occurs at a particular wavelength. The resonance parameter, either angle or wavelength, depends on the refractive index of the dielectric medium. In SPR biosensors, biomolecular recognition elements (e.g., antibodies) are covalently bound on the Au film surface. If the sample containing target molecules flow through the Au film surface, the target molecules will bind to the biomolecular recognition elements, leading to changes in the refractive index (RI) near the surface of the SPR biosensor. The ability to directly measure interactions in real time can allow researchers to quantitatively determine kinetic parameters, thermodynamics, and concentrations, or to qualitatively characterize relationships between ligands and analytes. The schematic diagram of this portable SPR bioanalyzer is shown in Figure 1; when the divergent polarized incident light beam has the correct incidence angle within TIR, reflection is decreased at the resonance angle. Obviously, the resonance condition depends on the angle of incidence, the wavelength of the light beam, and the dielectric functions of both the metal and the dielectric. The SPR shift in the resonance angle is directly proportional to changes in the refractive index at the Au surface. Based on this relationship, the SPR shift indicted with a sharp dip position is used as the assessment of the dielectric constant or refractive index changed on the Au surface. The photoelectric signals corresponding to the SPR shift were acquired via the linear charge-coupled device (CCD) array and used to establish a SPR response curve. In this experiment, the data digital-filtering algorithms were used to process the photoelectric signals from the linear CCD array.

A microfluidic cell, including a 3-way solenoid valve, was used to transfer samples over the biosensing chip. From Figure 1b, the homemade three-channel microfluidic cell (2.5 µL) was fabricated to irrigate three kinds of different samples controlled by the 3-way solenoid valve over the surface of the biosensing chip according to the practical demands. Fluidics are made up of 1/16 inch OD (outside diameter) fluorinated ethylene propylene tubing flowing into selected channels. The inlets and the outlets were formed by placing a thin PMMA (polymethyl methacrylate) plate over the PDMS (polydimethylsiloxane) gasket with six separate 1/16 inch holes drilled through, allowing access to each channel. Before the SPR bioanalyzer was used, the biomolecular recognition membrane was immobilized. The positions of the resonant dip on the linear CCD were shifted by changing the refractive index of the biological sample solution, which flowed through the microfluidic cell.

### 2.3. Immobilization of the Escherichia coli O157:H7 Antibody on the Au Film Surface

**Figure 2:**
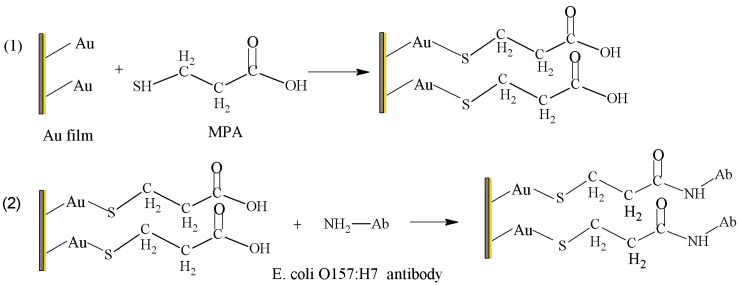
Schematic diagram of the procedure of *Escherichia coli* O157:H7 biomolecular recognition membrane. (1) A 3-MPA SAM was first attached to the biosensor surface; (2) The *E. coli* O157:H7 antibody molecules were immobilized on the sensor surface.

By using an ultrasonic cleaner, the Au film surface of the integrated SPR biosensor was cleaned with 4% SDS for 5 min, and it was then rinsed with deionized water and dried with N_2_ in advance. After that, the integrated SPR biosensor was firmly clamped with the Au film and then connected to the circuit board for the acquisition of SPR response signals. Generally, a self-assembled monolayer (SAM) was first attached on the surface of the sensor utilizing the covalent bonds between the thiol group and gold, forming a Au–S bond. In our experiment, a 3-MPA SAM was first attached to the biosensor surface to form the 3-MPA/Au SPR chip. Then, the carboxyl groups of 3-MPA molecules were released to interact with amino groups of the *E. coli* O157:H7 antibody. Thus, *E. coli* O157:H7 antibody molecules were easily immobilized on the treated sensor surface. The functionalization of the biomolecular recognition membrane is illustrated in Figure 2.

## 3. Results and Discussion

### 3.1. Evaluation of the Response Units from the SPR Biosensor for the Biomolecular Recognition Membrane

The detailed immobilization procedure of the *E. coli* O157:H7 antibody is described as follows. (1) A pH 7.4 PBS solution flowed to the surface of the Au film at a flow rate of 30 μL/min to obtain a constant baseline. Subsequently, 3-MPA (0.01 mol/L) was injected after the response signals began to emerge steadily with a line. Meanwhile, the response curve was formed by calculating the response signals from the circuit board, where the circuit board was used to acquire the SPR response signals and then display the signals on the touch-screen in real time. The PBS was blocked for a while and injected into the microfluidic cell again to keep the response signal still stable, which indicated that the carboxyl group layer was firmly immobilized on the Au film. (2) To activate the 3-MPA/Au SPR chip, the mixed solution of 0.4 mol/L EDC and 0.1 mol/L NHS with a mixture ratio of 1:1 (1:1, *V*/*V*) was injected. The activation time, which is defined as the time that it takes for the covalent attachment of the *E. coli* O157:H7 antibody on the surface of the EDC-NHS to activate 3-MBA/Au SPR chip, approximately one hour. (3) The 50-fold diluted antibody of *E. coli* O157:H7 was injected into the microfluidic cell, and the response signals were monitored in real time until the occurrence of the stable response signals. Afterwards, the PBS flowed through the surface of the SPR biosensor until the response signals steadily recurred, which indicated that the *E. coli* O157:H7 antibodies were firmly immobilized on the Au film. (4) A 1 mol/L ethanolamine solution was injected to block the carboxyl spots that were not bound with the *E. coli* O157:H7 antibodies on the surface of the Au film.

**Figure 3:**
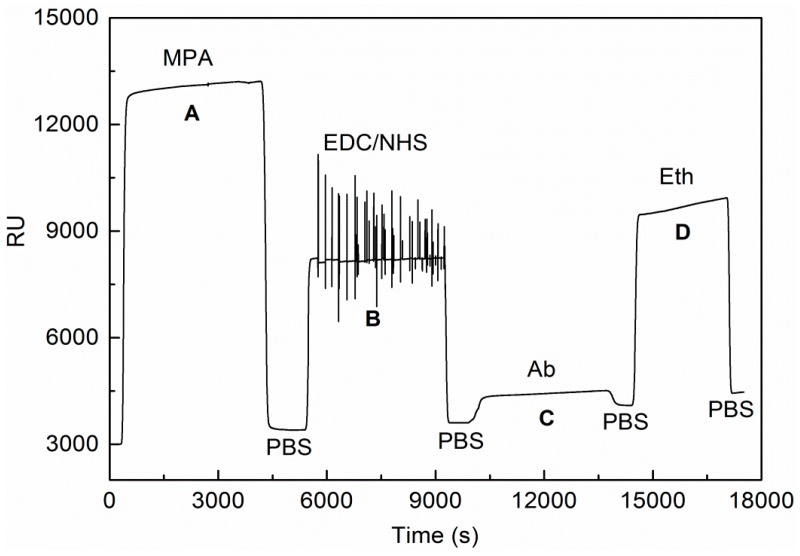
The response unit obtained from the functionalization of biomolecular recognition membrane.

The pH 7.4 PBS solution should be injected to obtain a constant baseline from the three SPR channels prior to the data acquisition of the biological samples. The response units (RUs) of the biomolecular recognition membrane are clearly shown in Figure 3. First, the MPA solution was injected to cause the –SH group to couple with the Au film deposited on the surface of the biosensor completely in order to immobilize the antibodies of *E. coli* O157:H7 (see Phase A in Figure 3). A background of 3000 was automatically set by the operating software after system initialization. A normal calibration method was used to convert the delta response unit ΔRU (ΔRU, the response unit of baseline, was subtracted by the current response unit, promptly), which was obtained from the PBS flowed through the Au film surface of the SPR bioanalyzer. After that, the resolution was improved by avoiding the calculation of the very small number. Here, it is equivalent to magnify the value of RU by 3000 times [26]. The delta response unit signal obtained from the binding of Au film with MPA was 457 RU after the Au film surface was washed by PBS. The mixture of EDC and NHS was injected through the Au film surface to activate the carboxyl groups of the self-assembled monolayer. During this activation process, a large amount of bubbles occurred, resulting in large fluctuations in the curve, as shown in Phase B in Figure 3. The response units (RUs) began to increase after the surface of the 50 nm Au film was washed with PBS. In Phase C in Figure 3, the *E. coli* O157:H7 antibody containing –NH_2_ groups was immobilized via covalent–CO–NH– amide bonding after the carboxyl groups on MPA were activated by the mixed solution of EDC and NHS. The RUs indicated with the baseline were 3602 before the antibodies of *E. coli* O157:H7 were fixed on the Au film surface of the integrated SPR biosensor, while the values of the RU reached 4461 at the equilibrium plateau after the *E. coli* O157:H7 antibodies were fixed on the Au surface of the biosensor; then, the values of the RU reached a stable level of 4098 after being washed by PBS. In this process, the RU increased after the immobilization antibodies of *E. coli* O157:H7 with an ΔRU of 496 RU. Sequentially, Eth was injected to seal off the carboxyl (see Phase D in Figure 3). It can be seen from Figure 3 that the ΔRU increased with the process of immobilization, which demonstrates that the biomolecular recognition membrane was prepared successfully.

### 3.2. Samples Analysis of E. coli O157:H7

**Figure 4:**
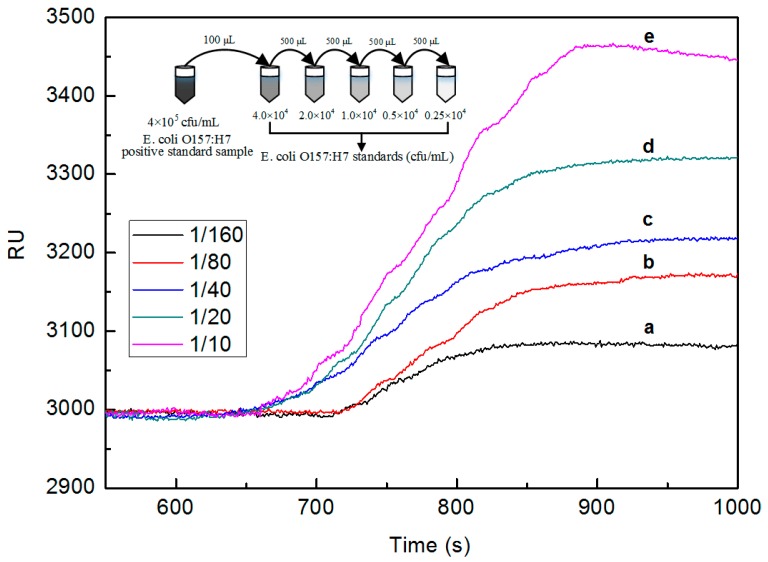
SPR sensorgrams generated from the detection of the positive *E. coli* O157:H7 sample.

For the investigation of sensitivity of the portable SPR bioanalyzer, the positive *E. coli* O157:H7 control sample was diluted 10-, 20-, 40-, 80-, and 160-fold with corresponding concentrations of 4.0 × 10^4^ cfu/mL, 2.0 × 10^4^ cfu/mL, 1.0 × 10^4^ cfu/mL, 0.5 × 10^4^ cfu/mL, and 0.25 × 10^4^ cfu/mL, respectively. The preparation process is illustrated in the inset of Figure 4. The *E. coli* O157:H7 antibody/3-MPA/Au SPR chip was regenerated via treatment with 0.1 M NaOH. The small changes in response unit resulted from the nonspecific binding of cells to the surface. This method completely regenerates the Au surface without affecting its surface properties and makes this process more cost-effective in decreasing the nonspecific binding. Thereafter, each of them flowed over the Au film surface of the integrated SPR biosensor. After the measurement on each different concentration, the non-specific reaction needed to be dissociated with PBS (pH = 7.4), and the surface also needed to be regenerated with NaOH (pH = 13) and acetonitrile solution. In Figure 4, the response values decrease when the dilution factor increases. Therefore, it is easy to distinguish the slight differences in terms of concentration. All this conforms to the law of direct detection.

**Figure 5:**
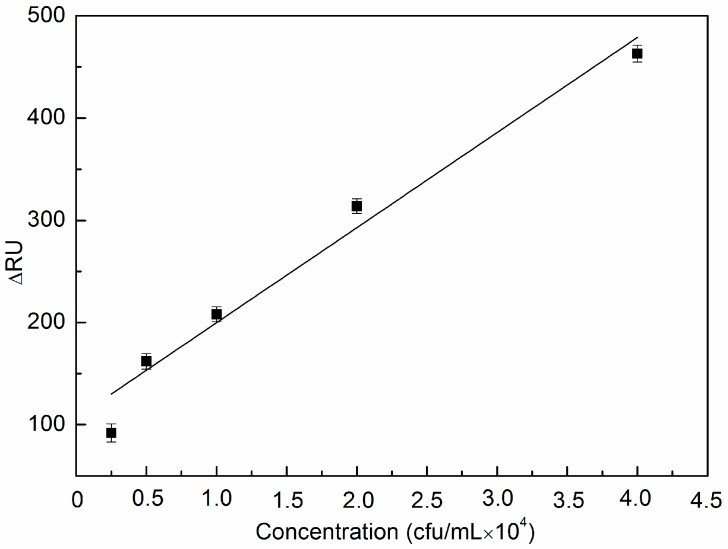
The relationship between different concentrations of *E. coli* O157:H7 and the SPR response signals.

The fitting curve (see Figure 5) was established based on the relationship between delta response units and different *E. coli* O157:H7 control samples, with known concentrations ranging from 0.25 × 10^4^ cfu/mL to 4 × 10^4^ cfu/mL. The relation is expressed as ∆RU = 0.0093 × *C* + 106.7265 with the correlation coefficient R of 0.982, where C is the concentration of *E. coli* O157:H7. Therefore, there is a positive proportional relationship between the value of the response signal ΔRU and the concentrations. The results show that a sensitivity of 2.5 × 10^3^ cfu/mL was obtained from the diluted positive control samples. Moreover, the limit of detection of 1.87 × 10^3^ cfu/mL was calculated from the empirical formula expressed as 3σ/*k*, where σ is the standard deviation, and *k* is the slope of the fitting equation.

### 3.3. Comparison with the E. coli O157:H7ELISA Kit

In order to compare the sensitivity of this portable SPR bioanalyzer, the commercial ELISA kit for on-site detection of *E. coli* O157:H7 was carefully administered. The designed use of an *E. coli* O157:H7 ELISA kit as an aid in the detection of *E. coli* O157:H7, following the same ELISA assay principles, is a qualitative, rapid test for the presumptive qualitative detection of *E. coli* O157:H7 from a variety of food matrices, including meat and dairy products. In this experiment, the *E. coli* O157:H7 positive sample from the ELISA kit was diluted 10-, 20-, 40-, 80-, and 160-fold with PBS. Under identical conditions, all dilution samples were measured with the portable SPR bioanalyzer and the *E. coli* O157:H7 ELISA kit. As shown in Table 1, the results from the portable SPR bioanalyzer are consistent well with the ELISA kit in the range of less than 40-fold dilutions. Moreover, the *E. coli* O157:H7 positive sample with as low as 160-fold dilutions can still be successfully detected using this portable SPR bioanalyzer. Therefore, the proposed portable SPR bioanalyzer here is fast and highly sensitive in comparison with the *E. coli* O157:H7 ELISA kit.

## 4. Conclusions

A portable SPR bioanalyzer for the sensitive detection of *E. coli* O157:H7 was systematically investigated based on the SPR biosensor by modifying the *E. coli* O157:H7 antibody on a layer of gold film. Based on the SPR bioanalyzer, a non-destructive, label-free, and real-time monitoring can be achieved. The positive *E. coli* O157:H7 control experiments were performed by having the diluted positive *E. coli* with 10-, 20-, 40-, 80-, and 160-fold solutions flow through the SPR bioanalyzer. To obtain the response values through monitoring binding response signal changes between the *E. coli* O157:H7 antibody and the *E. coli* O157:H7, the delta RU was substituted into the established standard concentration curve to obtain the concentration that needs to be detected. A linear relationship (the correlation coefficient R of 0.982) between the concentrations of *E. coli* O157:H7 and RU values was established. In comparison with the traditional molecular biology methods, the newly developed portable SPR bioanalyzer could be used as a stable, precise, and sensitive approach for quantifying *E. coli* O157:H7 in field.

## Figures and Tables

**Table 1:** Comparison of the sensitivity and specificity between the portable SPR bioanalyzer and the *E. coli* O157:H7 ELISA kit.

Diluted Factors	Original Positive Sample	10	20	40	80	160
SPR bioanalyzer	+	+	+	+	+	+
ELISA kit	+	+	+	+	−	−

+: indicating positive sign; −: indicating negative sign.
